# Primary Hydatid Cyst of the Kidney and Ureter with Hydatiduria in a Laboratory Worker: A Case Report

**DOI:** 10.1155/2012/596923

**Published:** 2012-10-23

**Authors:** Venkatesh Seetharam, Vinay Khanna, Padmapriya Jaiprakash, Kranthi Kosaraju, Joseph Thomas, Chiranjay Mukhopadhayay

**Affiliations:** ^1^Department of Urology, Kasturba Medical College, Manipal University, Karnataka 576104, India; ^2^Department of Microbiology, Kasturba Medical College, Manipal University, Karnataka 576104, India; ^3^Department of Pathology, Kasturba Medical College, Manipal University, Karnataka 576104, India

## Abstract

Hydatid disease is frequent in endemic regions and sheep farming areas. Most common localization of hydatid cyst occurs in liver followed by lungs. Renal hydatid cyst constitutes about 2–4% of all locations. We report a case of left renal hydatid from a laboratory technician admitted in a tertiary care hospital. There were few cases of renal hydatid disease reported in India among general population but to the best of our knowledge never reported from laboratory worker. The possibility of laboratory-acquired infection cannot be ruled out in this case due to lack of precautionary measures and containment facilities in resource-constrained setting.

## 1. Introduction

Hydatid disease or echinococcal disease is a parasitic disease that affects both humans and other mammals such as sheep, dogs, rodents, and horses. It is caused in humans by larval stage of *Echinococcus granulosus* complex, *E. multilocularis, E. vogeli,* or *E. granulosus*. Hydatid disease has a worldwide distribution, most of the cases are seen in sheep rearing areas with high prevalence in China, Central Asia, the Middle East, Eastern Africa, and some parts of South America. Echinococcus larvae are most commonly retained in the liver (60% cases) followed by lungs (20% cases). In the rest of cases, it gains access to the systemic circulation to reach other organs. Renal involvement is uncommon and seen in only 2% to 4% of cases [1]. Most patients with primary involvement of kidney remain asymptomatic for many years [2, 3]. 

## 2. Case Report

**Figure 1:**
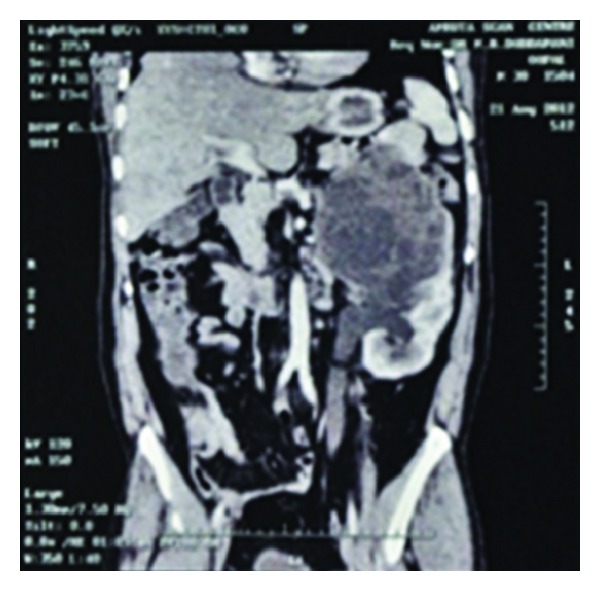
CECT abdomen revealed a 11 × 13 × 13 cm large cystic lesion in upper pole of left kidney.

A 37-year-old laboratory technician from north Karnataka presented with history of left flank pain and intermittent passage of small, pearly white balloon like grape size structures in the urine for 15 days. He gave a history of passage of similar structures in the urine few months ago. He also gave history of having been diagnosed to have a cyst in the left kidney on routine USG examination 18 years ago. However, he did not investigate it further since he was asymptomatic. On evaluation after admission to our institution, his routine investigations were within normal limits, chest and abdominal X-ray were normal. USG showed a well-defined cystic lesion occupying the upper and mid pole of left kidney measuring 12 × 12 cm with multiple cysts of varying sizes and hyperechoic stroma. CECT abdomen revealed a 11 × 13 × 13 cm large, cystic lesion (Figure 1). It had multiple daughter cysts giving rise to a spoke wheel pattern. The cyst was noted in the upper and mid pole of left kidney. It was associated with a moderate left hydroureteronephrosis. 

**Figure 2:**
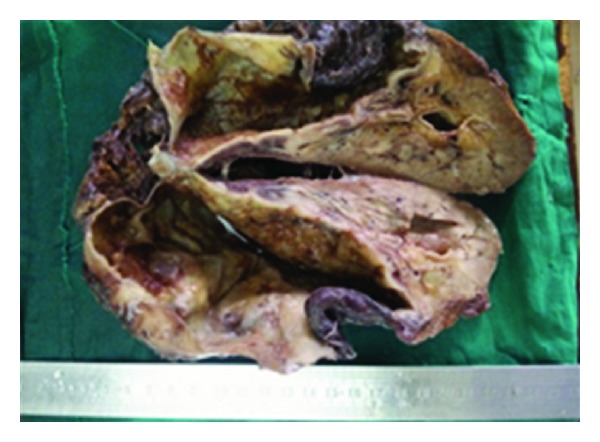
Cut section of kidney showing drained large unilocular cyst and multiple small cysts.

**Figure 3:**
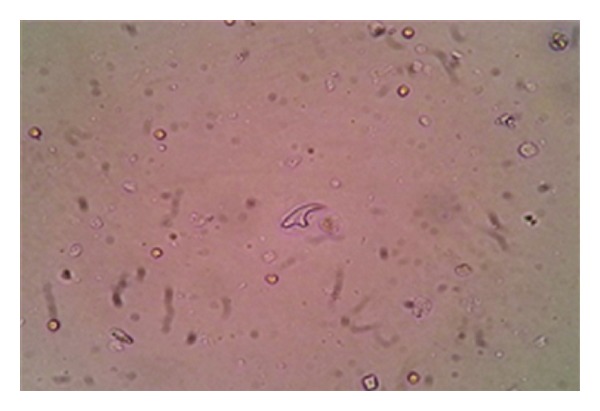
Saline wet mount showing hooklet of *Echinococcus* spp.

**Figure 4:**
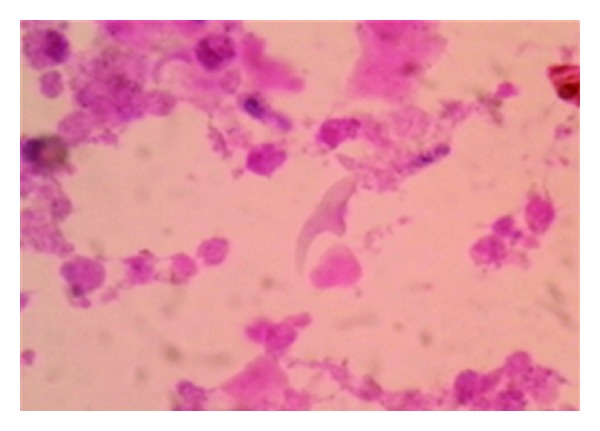
H&E stain showing hooklet of *Echinococcus* spp.

The patient underwent left nephroureterectomy through the flank extra peritoneal approach. Preoperatively, there was a large thick walled cyst in the upper and mid pole of the left kidney with a thick walled ureter. The resected specimen showed a large thick walled cyst with numerous daughters' cysts in the kidney and the upper half of the ureter. The histopathological examination (Figure 2) was consistent with left renal hydatid cyst with involvement of the ureter. Cyst fluid was sent for parasitological workup showed hooklets and protoscolices of echinococcus spp (Figures 3 and 4). Patient's serum also tested positive for IgG antibodies indicating lon-term infection. The patient received 3 weeks of albendazole preoperatively, and the same was continued for six week in the post-operative period. The postoperative period was uneventful. 

## 3. Discussion

Renal hydatid cysts are usually Multiloculated consist of single large cyst and smaller daughter cysts of varying sizes. multiple hydatid cysts in kidney were reported in literature [4]. Hydatid cyst wall is thickened with outer pericyte layer composed of fibrocollagenous lamellated chitinous layer and inner germinal layer with brood capsule and embedded hooklets. Renal pelvicalyceal system is dilated in most of the previously reported cases and microscopic hydatiduria is seen in 10–20% of renal hydatidosis [3].

In this case report, our patient was working as a laboratory technician for twenty years and given history of handling positive hydatid cyst samples in the past, it might be possible that he acquired infection in the laboratory if precautionary measures were not taken and containment measures were not in place. In the laboratory, the main source of echinococcus spp larvae and eggs are the positive patient tissue biopsy and stool specimens. Laboratory hazards can occur after accidental ingestion of eggs or mucocutaneous contact with fecal matter from experimentally infected animals. General containment requirements for handling hydatid disease are Biosafety level 2 practices and containment equipment for all activities involving the infective stages of the parasite and infectious body fluids or tissues, which are not available in most of the Indian laboratories due to resource constraints.

Our patient had a history of macroscopic hydatiduria. The diagnosis of renal hydatid disease is suggested by hydatiduria which is a pathognomonic feature but rarely documented. In this case, CT scan confirms the diagnosis of a left renal cyst with a moderate left hydroureteronephrosis. CT scan has advantage over USG as it can easily detect calcifications and daughter cysts and is more sensitive and accurate [5, 6]. The specific diagnosis of hydatid disease can be made by identifying protoscolices or hooklets in cyst fluid but usually fluid aspiration is not recommended due to risk of fluid leakage and anaphylaxis reaction. We had done a saline mount, modified Baxby staining procedure and Haematoxylin and eosin staining on cyst fluid for better visualization of hooklets [7]. The ruptured cyst causes significant stimulation of antibodies irrespective of the location [8]. IgG is found to persist for many years in these cases. In our case, patient has positive serological response to disease in the form of IgG antibodies, which could be due to chronic history.

Nephrectomy (25% cases) remains the treatment of choice for complicated renal hydatid cysts (e.g., those communicating with the ureter and seeding in multiple areas) and completely destroyed kidney where partial removal can lead to relapse and incomplete cure. In 75% of the cases, kidney sparing surgery can be done which consists of the cystectomy with ablation of the hydatid membrane and of small vesicles [4, 5]. There are few reports of laparoscopic removal of renal hydatid but chances of cyst rupture, dissemination, and incomplete removal of the hydatid cyst are quite high [9]. Albendazole is medical treatment of choice in disseminated hydatid disease, localized disease with poor surgical risk, ruptured cysts, and significant intraoperative spillage. Pre- and post-operatively use of albendazole decreases the cyst wall tension thus reducing the risk of spillage during surgery and prevent the chance of anaphylaxis [10].

## 4. Conclusion

Laboratory-associated hydatid disease transmission, although rare, should be suspected in patients working in laboratories and presenting with mass lesions in relevant organs especially in endemic regions and resource-constrained setup. These mass lesions should be confirmed with parasitological and radiological workup, followed by the definitive treatment and prevention. It is highly recommended to follow stringent work place infection control practices while handling suspected hydatid disease samples and it is better to screen laboratory workers for echinococcosis to prevent spread of the disease.
